# 1, 25(OH)2 D3 Induces Reactivation and Death of Kaposi’s Sarcoma-Associated Herpesvirus of Primary Effusion Lymphoma cells

**DOI:** 10.1038/s41598-017-12676-x

**Published:** 2017-09-29

**Authors:** Amit Kumar, Suchitra Mohanty, Piyanki Das, Sushil Kumar Sahu, Shanmugam Rajasubramaniam, Tathagata Choudhuri

**Affiliations:** 1 Infectious Disease Biology, Institute of Life Sciences, Bhubaneswar, India; 2 Department of Biotechnology, Regional Medical Research Centre for Tribals, Jabalpur, India; 3 Department of Biotechnology, Siksha Bhavana, Visva Bharati, Santiniketan, Bolpur, India

## Abstract

Kaposi’s sarcoma associated herpesvirus (KSHV) a gammaherpesvirus establishes perennial latency in the host with periodic reactivation. Occasionally change in the physiological condition like hypoxia, host cell differentiation can trigger the lytic switch and reactivation of the virus. The biologically active form of 1, 25(OH)2 D3 plays a critical role in the regulation of various physiological processes (e.g. regulation of mineral homeostasis and control of bone metabolism). Apart from its role in host physiology, 1, 25(OH)2 D3 has been implicated as a potential agent for the prevention and/or treatment of many a tumors. Here we show that 1, 25(OH)2 D3 induces both death of Kaposi sarcoma associated herpesvirus infected PEL cells and KSHV replication. 1, 25(OH)2 D3 mediated inhibition of proliferation was associated with apoptosis of the PEL cells, and virus reactivation. In addition, p38 signalling is required for KSHV reactivation. Furthermore, treatment of PEL cells with p38 inhibitor abrogated the expression of ORF57, thus blocking lytic switch. Furthermore, silencing of VDR resulted in reduced ORF57 expression compared to the control cells, signifying the potential role of 1, 25(OH)2 D3 in KSHV reactivation. Thus, our studies have revealed a novel role of 1, 25(OH)2 D3 in the regulation of KSHV reactivation and PEL cell death.

## Introduction

Kaposi’s sarcoma associated herpesvirus (KSHV) is a DNA tumor viruses belonging to a member of gammaherpesvirus family and is associated with Kaposi sarcoma (KS), Primary effusion lymphoma (PEL) and a subtype of multicentric castleman disease (MCD)^1–4^. KSHV like other herpesvirus exhibits two different life cycles, latent and lytic. During latent infection, only a subset of genes are expressed, which enable KSHV to evade immune system and promote viral persistence^5–7^. While lytic cycle, lytic proteins are expressed in an ordered cascade to produce virons for their efficient propagation and transmission^8,9^. Studying induction of lytic switch provides an opportunity to understand the infection and pathogenesis of KSHV associated diseases. The switch from latent to lytic replication is an active area of research and has contributed to a large extent information about the cellular factors with possible roles in reactivation mechanisms. However the regulation of KSHV pathogenesis by metabolic pathways is still only sparsely understood.

Primary effusion lymphoma (PEL) is a rare HIV-associated non-Hodgkin’s lymphoma (NHL), resembles a transformed post-germinal center (GC) B cell^10–12^. PEL typically presents with lymphomatous body cavity effusions in the absence of solid tumor masses harbouring KSHV episomes and arise preferentially within the pleural or peritoneal cavities of approximately 4% of all HIV associated NHLs^13–15^. KSHV infection of PEL cell is predominantly latent, which makes PEL cells an ideal cell lines to study two phases of its life cycle^16^. Therapeutic induction of virus replication is necessary to target and eliminate KSHV associated tumor cells. Earlier studies have attempted induction of KSHV reactivation with a different compounds or drugs^17–19^.

Vitamin D3 was originally identified as a key regulator of bone metabolism and calcium homeostasis^20^. Most of the biological action of 1, 25(OH)2 D3 are exerted through nuclear receptor vitamin D receptor (VDR)^21^. Apart from bone metabolism and calcium homeostasis, 1, 25(OH)2 D3 has been shown to be involved in the control of angiogenesis, apoptosis, Immunomodulation, growth and differentiation of many cell types, including lymphoma cells^22–26^. VDR expression is reported in many cancers types including breast, prostrate, pancreas, colon, leukaemia’s and lymphomas^27–32^. Exposure of these cells to 1, 25(OH)2 D3 induces apoptosis in cells. However, studies are lacking on the role of 1, 25(OH)2 D3 in viral pathogenesis, only very few studies have indicated that vitamin D3 deficiency may confer increased risk of influenza and respiratory tract infection^33,34^. *In vitro* studies have demonstrated the effect of 1, 25(OH)2 D3 in susceptibility and control of HIV infection^35^. Furthermore, pre-treatment of human monoblastoid U937 cell line and monocyte derived macrophages in cell culture model of HIV infection have demonstrated anti-viral effects^36^. However, the underlying mechanism or pathways involving these functions is unclear, due to varied activities and functions. In addition, it remains to be identified whether 1, 25(OH)2 D3 is protective or pathogenic in cases of viral infection.

Effect of 1, 25(OH)2 D3 on downregulation of NF-κB pathway in endothelial cells transformed by Kaposi sarcoma associated herpes virus G protein coupled receptor is known^37^. Further, it has been shown that 1, 25(OH)2 D3 also has anti-proliferative effect on KSHV GPCR transformed endothelial cells^38^. Gene expression profiling of PEL cells have demonstrated that VDR is highly expressed in PEL cells as compared to normal B and T cell lymphoma and their sensitivity to vitamin D analogue EB1089, implicates a role for VDR in KSHV pathogenesis^11^. In view of these facts, the current investigations were taken up to dissect the mechanism (s) of action of 1, 25(OH)2 D3 on PEL cells, in particular its effect on apoptosis and reactivation.

## Material and Methods

### Cells and Reagents

PEL cells (JSC-1 and HBL-6) were kindly provided by Erle Robertson (University of Pennsylvania). These cells were cultured in RPMI 1640 supplemented with 10% foetal bovine serum glutamine (300 mg/mL) and streptomycin (100 mg/mL) and penicillin (100 U/mL) under 5% CO2 at 37 °C. 1, 25(OH)2 D3 was purchased from Sigma-Aldrich and was reconstituted in 90% ethanol and stored at −80 °C in an inert atmosphere in the dark. In all experiments, equal amount of 90% ethanol were added to control cultures. Pan caspase inhibitor Z-VAD-FMK was purchased from R&D system. FITC annexin V apoptosis detection kit was purchased from BD Biosciences, SB203580 (p38 inhibitor) and PD98059 (ERK inhibitor) were purchased from InvivoGen, phorbol 12-myristate 13-acetate, sodium Butyrate and MTT reagent were purchased from Sigma-Aldrich.

### Cell viability assay

All cells were plated in 96 well culture plate in complete medium at a density of 5 × 10^4^ cells per well and treated with or without increasing concentration of 1, 25(OH)2 D3(10, 50, 100, 200 nM). The plates were incubated at 37 °C, 5% CO2, for 24, 48 and 72 hours, respectively. Then, MTT solution (10 μL) for a total volume of 100 μL was added in every well and incubated for 4 hours at 37 °C with 5% CO2. Subsequently, MTT-containing medium was removed gently and replaced with DMSO (100 μL per well) and absorbance was obtained at 570 nm on a microtiter plate reader.

### shRNA mediated VDR knockdown

To knock down VDR expression, two validated lentiviral constructs expressing small hairpin RNA (shRNA) sequences to targeting 2 different regions of the human VDR transcript were used. The constructs were obtained from (Sigma-Aldrich). Details of the clones and target sequences are given in Table 1. Lentiviral particles were prepared using standard protocols, resuspended in serum-free media and used to transduce JSC-1 cells. After 48 h, stably transduced cells were selected for puromycin resistance (2.5 μg/mL) for 20 days.

**Table 1:** Short-hairpin RNA clones used to silence VDR function.

TRC clone ID	Target sequence	Location on VDR transcript (GI: 340202)
TRCN0000019505	GTCATCATGTTGCGCTCCAAT	923–943
TRCN0000019506	CCTCCAGTTCGTGTGAATGAT	578–598

### Quantitative Real Time RT-PCR (qRT-PCR)

Total RNA was extracted from cells using TRIzol reagent (Invitrogen, Life Technologies, USA) as per manufacturer’s instruction, followed by treatment with DNase 1. One microgram total RNA was reverse transcribed using cDNA synthesis kit (Thermo Fischer, USA). Syber green PCR was performed using primer specific for KSHV ORF57, RTA and the human GAPDH gene. Sequence of primers are mentioned in Table 2.

**Table 2:** List of qRT-PCR primers.

ORF57	F-5′-TGGACATTATGAAGGGCATCC-3′
	R-5′-CGGGTTCGGACAATTGCT-3′
RTA	F-5′-CAGACGGTGTCAGTCAAGGC-3′
	R-5′-ACATGACGTCAGGAAAGAGC-3′
LANA	F-5′-CATACGAACTCCAGGTCTGTG-3′
	R-5′-GGTGGAAGAGCCCATAATCT-3′
K8.1	F-5′-AAAGCGTCCAGGCCACCACAGA-3′
	R-5′-GGCAGAAAATGGCACACGGTTAC-3′
GAPDH	F-5′-CCACATCGCTGAGACACCAT-3′
	R-5′-TTCCCGTTCTCAGCCTTGAC-3′

### Cell death assay using flowcytometry

JSC-1 and HBL-6 cell were treated with 10 nM of 1, 25(OH)2 D3 for 48hr. The cells were harvested and the percentage of cells undergoing apoptosis was measured by flow cytometry after staining with fluorescein-conjugated Annexin V and propidium iodide (BD Pharmingen, USA), according to the manufacturer’s recommendation The effect of 1, 25(OH)2 D3 (24 h post treatment) in presence or absence of Z-VAD-fmk was examined. The stained cells were acquired using BD LSRFortessa and analysed with BD FACS Diva software.

### Western blot analysis

Cells were lysed in modified RIPA buffer containing 150 mM NaCl, 1% NP-40, 50 mM Tris-HCl (pH 8), 0.5% deoxycholic acid, 0.1% SDS, 1% Triton X-100, protease and phosphatase inhibitors. Lysates were placed on ice for 45 minutes and then clarified by centrifugation. Supernatants were removed and total protein measured by Bradford assay. Forty microgram of protein lysate per lane was electrophoresed on 12% SDS-PAGE and transferred to nitrocellulose membranes. The membranes were blocked for 1 h in TBST blocking solution, containing 5% bovine serum albumin and then incubated with a primary antibody overnight at 4 °C. The membranes were washed at least 3 times with each wash for 10 min with washing solution (TBS and 0.1% Tween 20) and incubated for 45 min with appropriate horseradish peroxidase-conjugated secondary antibodies. The washed membranes were developed using ECL Blotting Substrate (Thermo Scientific). The β-actin,VDR, ORF57, K8α and ERK antibodies were purchased from Santa Cruz Biotechnology. The phospho-p38 mitogen-activated protein kinase (MAPK), LANA and caspase-3 antibodies were purchased from Imgenex.

### Viral Load Assay

For intracellular Viral load assay, DNA was isolated using Gene elute mammalian genomic DNA isolation kit according to manufacturer’s instructions (Sigma-Aldrich) and KSHV replication was determined by qPCR using SYBR green PCR master mix (Agilent technology, USA). KSHV ORF57 gene expression compared to vehicle controls. KSHV ORF57 gene expression was compared to vehicle controls. The qPCR reactions were carried out using LC480 machine (Roche life science, USA). Relative fold expressions were determined by the ∆∆CT method^39^.

### KSHV infection assay

JSC-1 cells were treated with 1, 25(OH)2 D3, vehicle, or TPA for 48 h. Supernatants were harvested and added to confluent monolayers of uninfected 293 cells in a 24 well dish. Polybrene (8 µg/mL) was added to each well and the plate was spinoculated at 2500 rpm for 2 h at 26 °C as previously described^40^. Ninety-six hours post-infection, intracellular viral loaded was determined by real time PCR. Furthermore, infection was validated by checking the expression of LANA by western blot.

## Results

### Antiproliferative effect of 1, 25(OH)2 D3 in PEL cell lines

**Figure 1:**
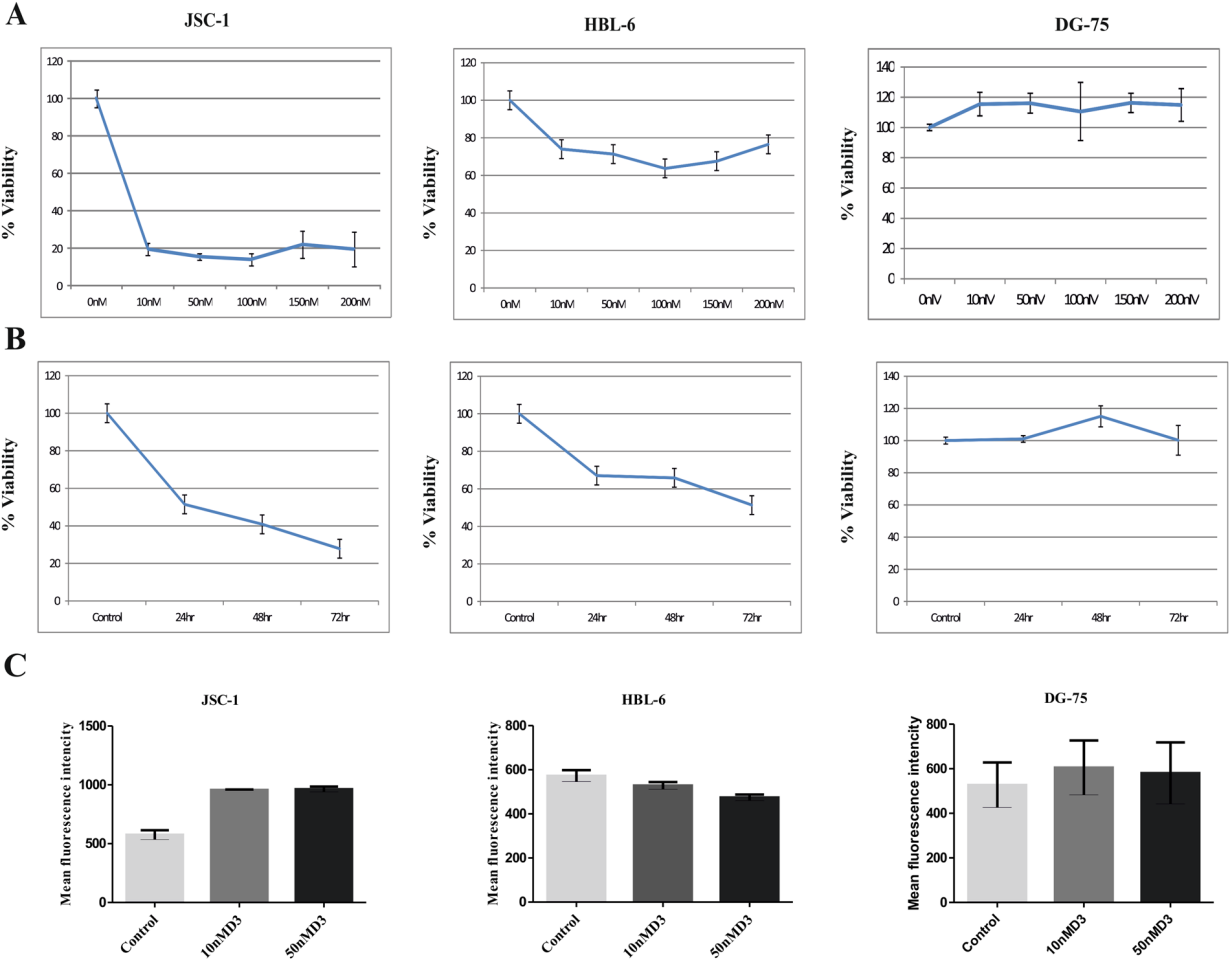
1, 25(OH)2 D3 induced loss of viability in PEL cell lines. (**A**) JSC-1, HBL-6 and DG-75 were treated for 48 h with different 1, 25(OH) 2 D3 concentrations (10, 50,100,150 and 200 nM) or (**B**) for different times (24, 48 and 72 h) with 10 nM of 1, 25(OH)2 D3, and cell viability was measured by MTT assays. The data shown here are representative of 3 independent experiments. Error bars indicate standard deviations. (**C**) JSC-1, HBL-6 and DG-75 cells were assessed for expression of vitamin D receptor by immunofluorescent flow cytometric assay.

The demonstration of VDR expression in diverse tumors and cancers has emphasized that the effect of 1, 25(OH)2 D3 is not limited to VDR expression only but also display a range of antiproliferative activities. JSC-1, HBL-6 and DG-75 cells were exposed to different 1, 25(OH)2 D3 concentrations, (0 to 200 nM), for 48 h and cell viability was tested. 1, 25(OH)2 D3 induced a dose-dependent loss of viability in JSC-1 and HBL-6 PEL cells as compared to control cells DG-75 (Fig. 1A). A time-kinetic investigation showed that 1, 25(OH)2 D3 treatments (10 nM) increased cell death between 24 and 48 h in PEL cell lines but not in DG-75cells (Fig. 1B). In general, JSC-1 cells showed higher sensitivity to 1, 25(OH)2 D3 as compared to HBL-6 cells. This varied sensitivity to the 1, 25(OH)2 D3 treatment correlated to the level of VDR expression (Fig. 1C). The expression of VDR was slightly upregulated by treatment with 1, 25(OH)2 D3. Flowcytometry showed higher VDR expression in JSC-1 and DG-75 cells with 1, 25(OH)2 D3 (Fig. 1C). In contrast, VDR levels were lower in HBL-6 cells (Fig. 1C). Collectively, the above data shows that the PEL derived cell lines are remarkably sensitive to 1, 25(OH)2 D3 induced growth inhibition.

### 1, 25(OH)2 D3 induces caspase-3 dependent cell apoptosis

**Figure 2:**
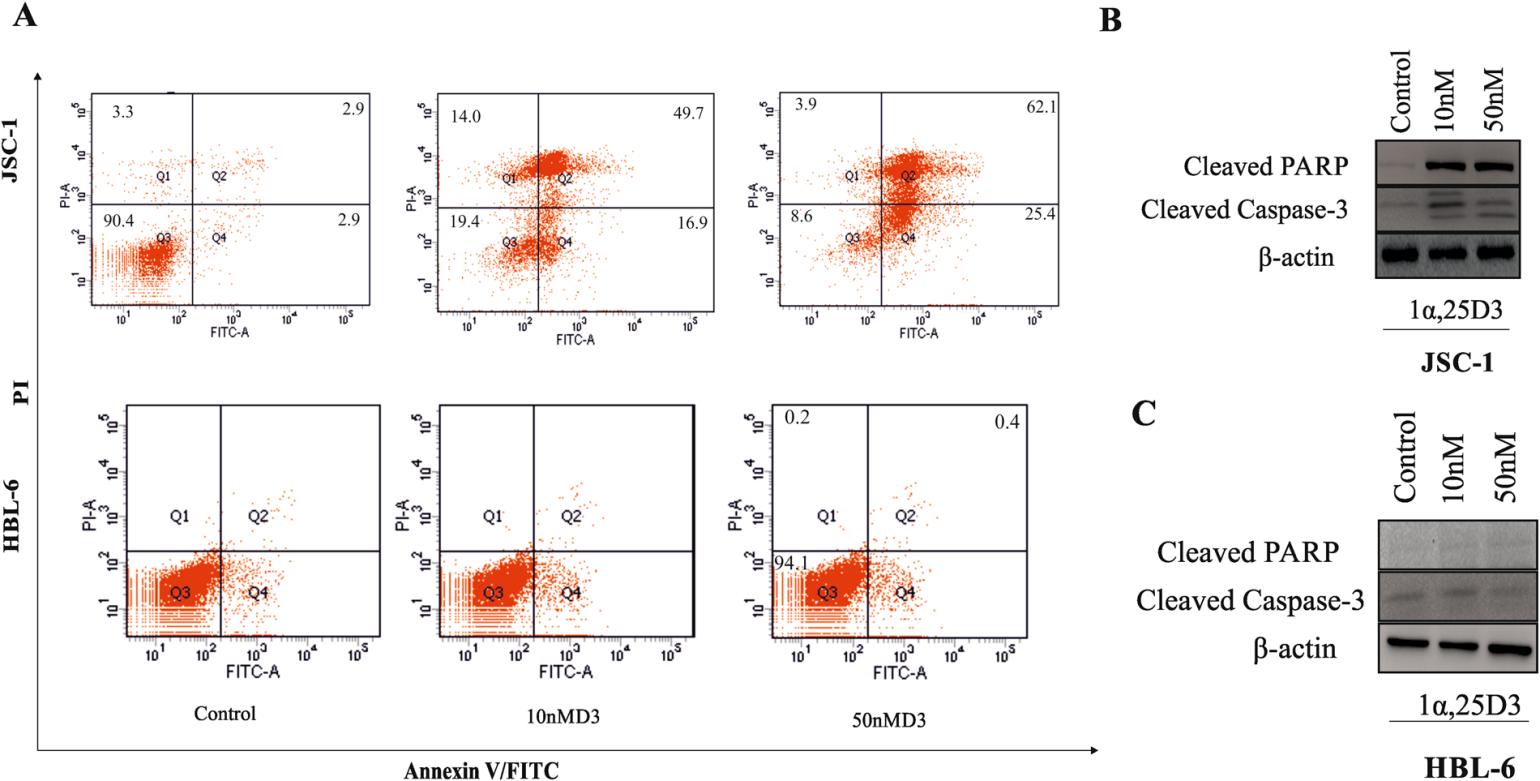
1, 25(OH)2 D3 induces apoptosis of PEL cells. PEL cells were cultured in the presence or absence of 1, 25(OH)2 D3 (10 nM). (**A**) After 48 h in culture, cells were washed and stained with Annexin V and PI and analyzed by flow cytometry. The numbers in the lower *right quadrant* represent the percent of apoptotic cells in culture. (**B** and **C** *)*. Cleaved caspase-3, and cleaved PARP expressions were detected by Western blot in JSC-1 and HBL-6 cells after treatment with 1, 25(OH)2 D3. β-actin was used to normalize protein loading.

**Figure 3:**
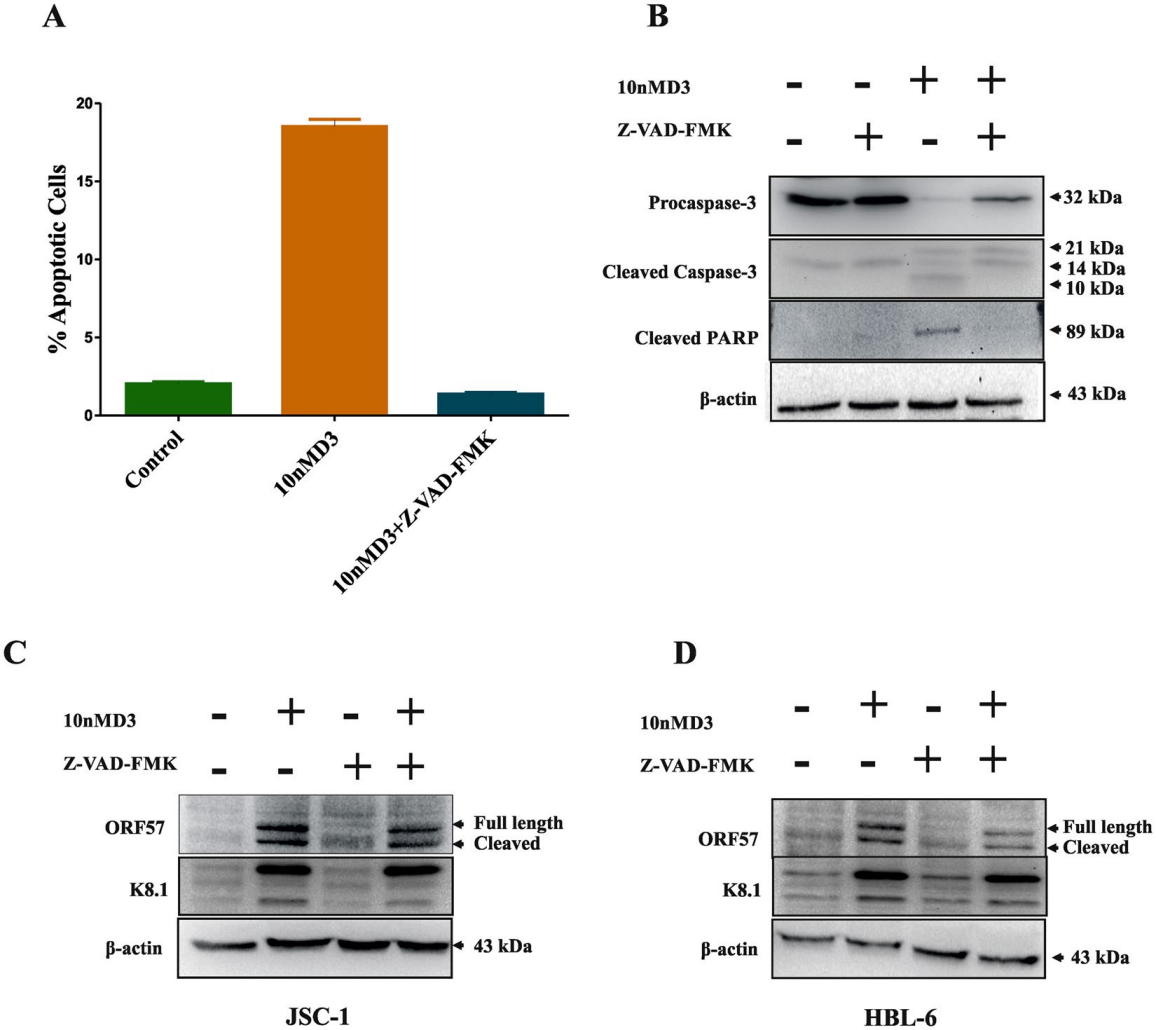
Effects of caspases inhibition by Z-VAD-FMK on apoptosis in PEL cells. JSC-1 cells were pre-treated with the caspase inhibitor Z-VAD-FMK (20 µM) for 2 h, followed by treatment with 10 nM 1, 25(OH)2 D3 for 24 h. (**A**) Cells were stained with Annexin V/PI, and apoptosis was determined using flow cytometry. Values for cells treated with DIM and Z-VAD-FMK were significantly reduced as compared to treatment with 1, 25(OH)2 D3 alone. Total protein extracts were prepared and subjected to Western blot assay for cleaved PARP and cleaved caspase-3 (**B**). Cell lysates collected 24 h after induction with the 1, 25(OH)2 D3 (10 nm) were immunoblotted with anti-ORF57 and anti K8.1 antibody. Effect of Caspase inhibitor on the expression of ORF57 or K8.1 in cells treated with 1, 25(OH)2 D3 alone or both 1, 25(OH)2 D3 and Z-VAD-FMK (**C** and **D**). β-actin was used to normaliz protein loading.

To determine whether the inhibitory effects of 1, 25(OH)2 D3 on viability was associated with the induction of apoptosis, we evaluated the percentage of apoptotic cells by annexinV/PI staining. 1, 25(OH)2 D3 treated JSC-1 cells showed significantly higher percentage of apoptotic cell after 48 h (Fig. 2A). On the other hand, HBL-6 did not show any significant change in apoptosis (Fig. 2A). Simultaneously we also evaluated the effect of 1, 25(OH)2 D3 on the expression of pro and anti-apoptotic proteins. Significant increase in the cleaved PARP and caspase-3 was observed in JSC-1 cells (Fig. 2B). However, only a modest change in the level of these proteins was found in HBL-6 cells (Fig. 2C) indicating higher sensitivity of JSC-1 cells to 1, 25(OH)2 D3. Thus suggesting that VDR contributes to the cellular apoptosis induced by 1, 25(OH)2 D3 in PEL cells. Further to prove the involvement of caspases in 1, 25(OH)2 D3 induced cell death pan-caspase inhibitor Z-VAD-FMK was used. Pan Caspase inhibitor Z-VAD-FMK completely repressed cellular death as evidenced by reversal of capspase-3 and cleaved PARP to the basal levels (Fig. 3A and B). Treatment of cells with z-VAD-FMK efficiently blocked cleavage of PARP-1 and Caspase-3 in 1, 25(OH)2 D3 treated JSC-1 and HBL-6 cells. Importantly however, it did not prevent expression of lytic protein ORF57 and K8.1 in 1, 25(OH)2 D3 treated cells (Fig. 3C and D).

### 1, 25(OH)2 D3 induces lytic gene expression and virus production in KSHV infected PEL cell lines

**Figure 4:**
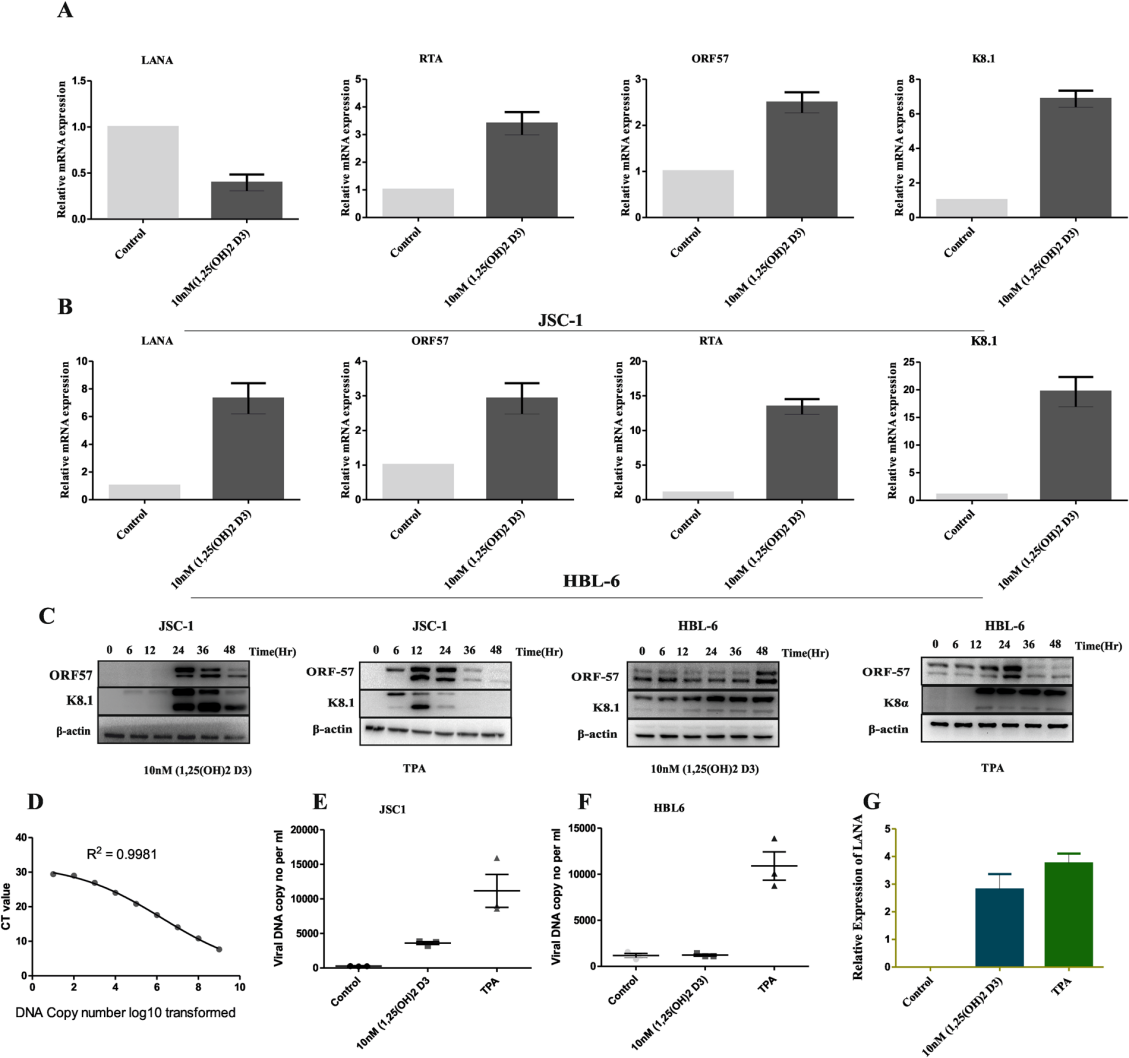
1, 25(OH)2 D3 induces viral lytic gene expression in KSHV-infected PEL cells. JSC-1 (**A**) and HBL-6 (**B**) cells were treated with either 1, 25(OH)2 D3 (10 mM), or vehicle for 48 h, then viral latent (LANA) and lytic gene (RTA, K8.1, ORF57) transcripts were quantified using qRT-PCR. Error bars represent the S.E.M for three independent experiments. (**C**) JSC-1 and HBL-6 cells were treated as above and TPA (20 ng/mL used as positive control) and then the expression of viral lytic protein K8.1 and ORF57 was checked by western blotting. β actin was used as loading control. (**D**) Standard curves for the quantification of Viral DNA (KSHV), Dilution series containing between 10^1^ and 10^9^ copies of LANA plasmid were used as quantification standards. (**E** and **F**) Absolute copy numbers of viral DNA derived from standard curve was plotted for controls and 1, 25(OH)2 D3 treated JSC-1 and HBl-6 cells (**G**) JSC-1 cells were treated by vehicle, 1, 25(OH)2 D3 (10 mM), and TPA (20 ng/mL as a positive control), respectively, for 48 h, then the virions were collected as described in Methods, followed by infection of 293 cells. LANA transcripts from each group were quantified by qRT-PCR. Error bars represent the S.E.M. for 3 independent experiments.

To determine whether VDR activation induces expression of KSHV lytic genes and virus production in 1, 25(OH)_2_ D3 treated PEL cells, JSC-1 and HBL-6 cells were treated with cognate 1, 25(OH)_2_ D3 for 48 h and monitored for the expression of LANA, RTA, ORF57 and K8.1. Significant increase in RTA, ORF57 and K8.1 were observed upon addition of 1, 25(OH)2 D3 (Fig. 4A and B). We then tested whether the increase in the expression of lytic genes also correlated to progeny virus production. For this latently infected JSC-1 cells were used, wherein cell-free virus was isolated from JSC-1 supernatants 2 days post 1, 25(OH)2 D3 treatment,.Viral DNA was extracted, and viral genome copy number determined by qPCR(4D,E and F). Figure 4G clearly demonstrates that 1, 25(OH)2 D3 induced virus production when compared to the controls. However, KSHV reactivation was comparatively lower in 1, 25(OH)_2_ D3 treatment than in the positive control, TPA (Fig. 4G). To validate further, a time course treatment of JSC-1 and HBL-6 cells with 1, 25(OH)2 D3 or a combination of both TPA and sodium butyrate (NaB) as a potent positive control for KSHV reactivation was performed [0,6,12,24,36 and 48 h] (Fig. 4C). Immunobloting, for expression of ORF57 and K8α showed lytic replication at the 24 h and 36 h with peak activation at 24 h (Fig. 4C). These results indicate that 1, 25(OH)2 D3 induces expression of lytic genes and progeny virus production.

### 1, 25(OH)2 D3 activates KSHV replication involving MAPK signalling pathway

**Figure 5:**
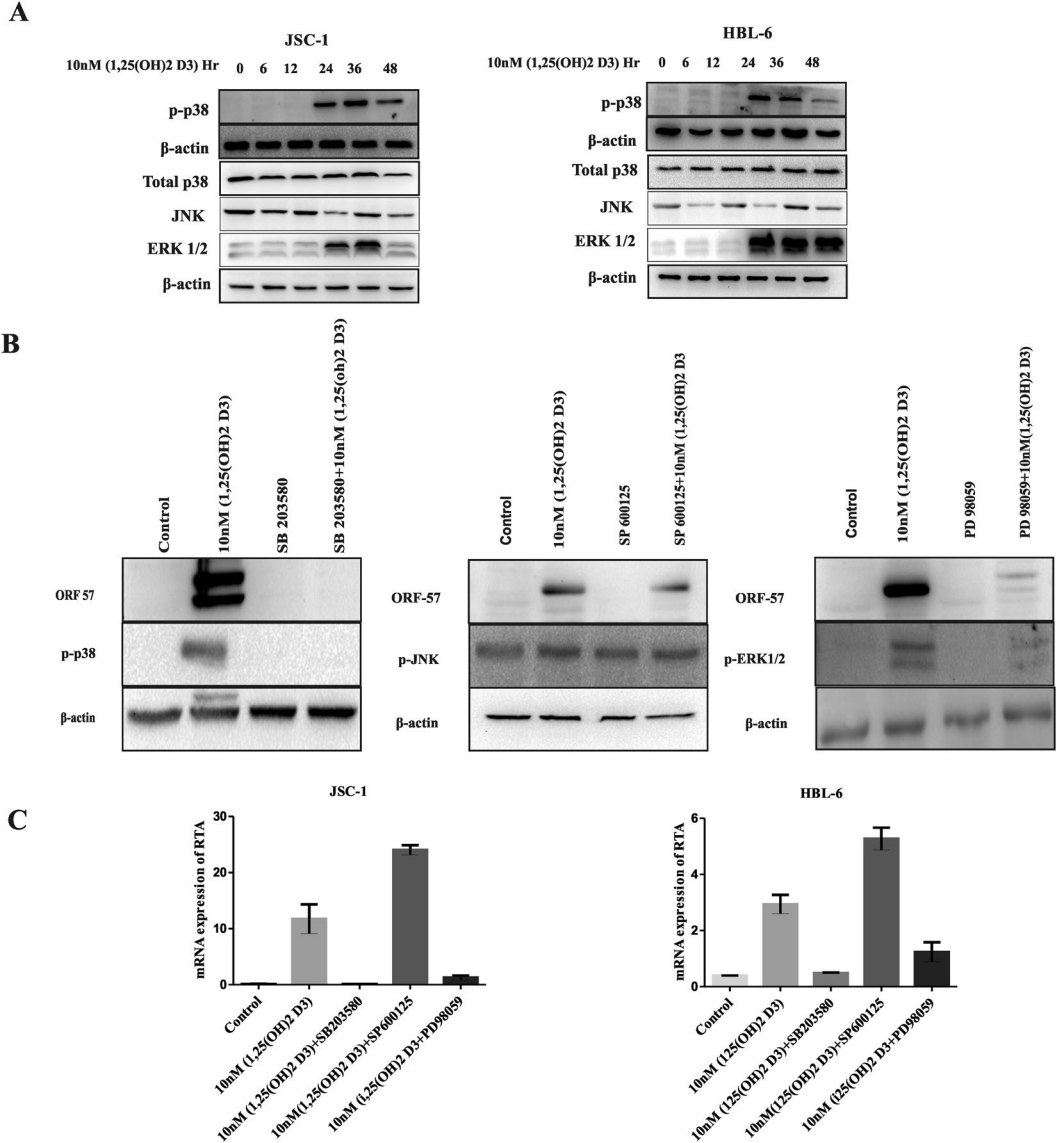
Induction of MAPK signalling by 1, 25(OH)2 D3. 1, 25(OH)2 D3 induces MAPK signalling pathway in PEL cells. (**A**) Western blotting with anti-phospho-p38, p-JNK,and anti p-ERK and anti β-actin antibodies of cell lysates of JSC-1 and HBL-6 cells treated with 10 nM D3 for 0 to 48hr showed phosphorylation of p-38 and induction of MAPK activity. (**B**) Cells were induced with 1, 25(OH)2 D3 in the presence of inhibitor of p38 (SB203580), ERK (PD98059) and JNK (SP600125), and analysed for the expression of ORF57 protein at 24 h. (**C**) Total RNA was extracted and subjected to qRT-PCR with the indicated primers to examine RTA expression in JSC-1 and HBL-6 cells. All the experiments were carried out three times, each with three replicates.

To gain insights into the mechanism underlying the role of 1, 25(OH)2 D3 in the induction of reactivation, we explored cellular signalling pathways that may mediate KSHV reactivation downstream of VDR signalling. Previous studies have shown that the mitogen-activated protein kinase (MAPK) signalling pathways play important roles in KSHV reactivation induced by phorbol esters, Ras and oxidative stress^41–44^. We therefore examined whether MAPK signalling is required for KSHV reactivation induced by 1, 25(OH)2 D3. PD 98059, a specific inhibitor of ERK, and SB 203580, a specific inhibitor of p38 MAPK, significantly inhibited KSHV reactivation induced by 1, 25(OH)2 D3, as indicated ORF57 protein levels (Fig. 5B). In contrast, the JNK inhibitor SP 600125 did not significantly affect KSHV reactivation induced by 1, 25(OH)2 D3 (Fig. 5B). Since 1, 25(OH)2 D3 upregulated RTA transcription (Fig. 4A and B), we further tested whether ERK or p38 signaling is involved in this upregulation. Both PD 98059 and SB 203580 inhibited RTA upregulation but not SP 600125 (Fig. 5C). Thus, ERK and p38 signalling are involved in the upregulation of RTA and KSHV reactivation downstream of VDR signalling.

### Effect of VDR knockdown on 1, 25(OH)2 D3 mediated KSHV Reactivation

**Figure 6:**
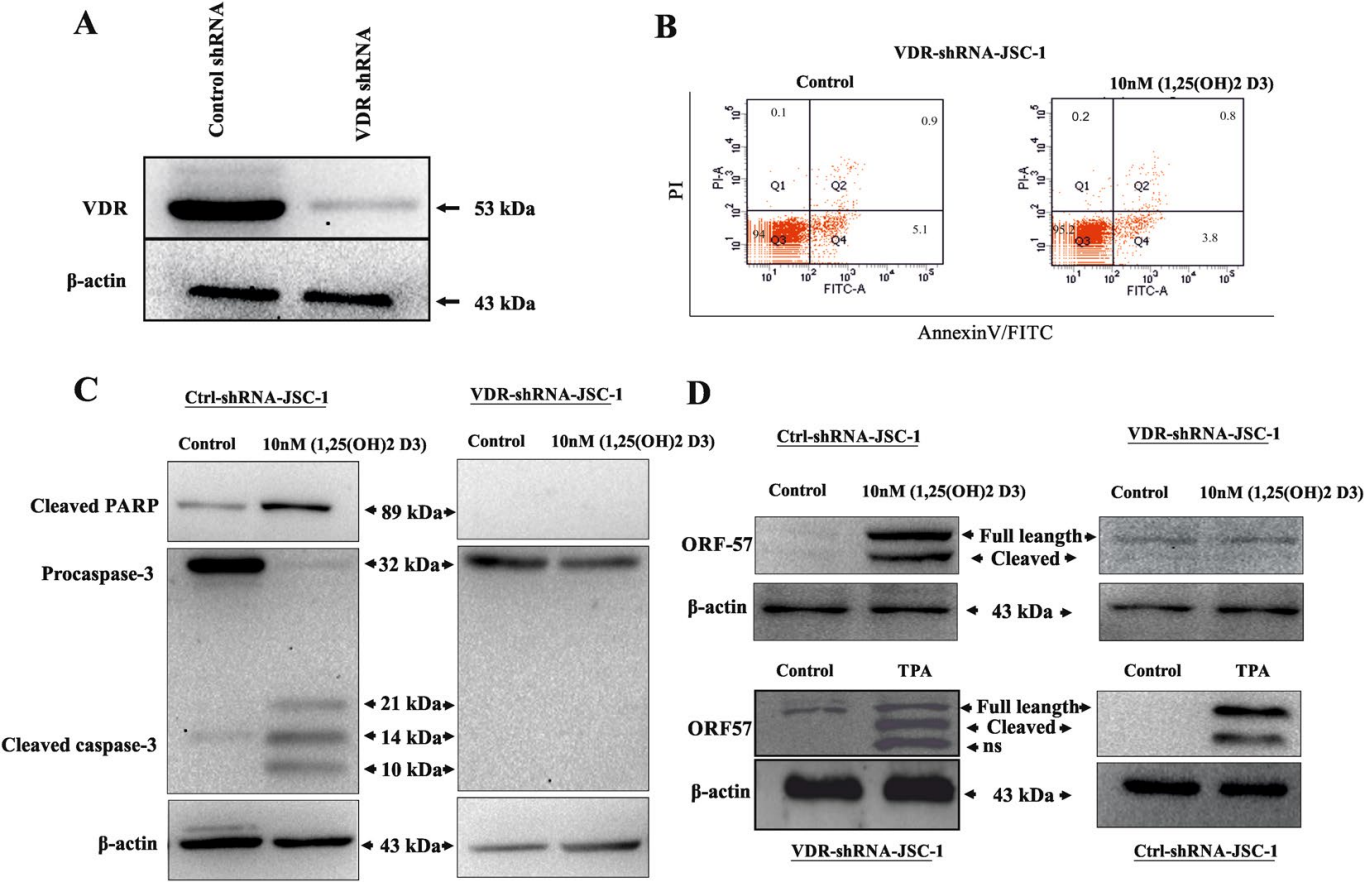
VDR knockdown inhibits KSHV reactivation. JSC-1 cells were transduced with lentiviral particles containing either scrambled shRNA or shRNAs directed against the human VDR transcript. (**A**) Western blot analysis of stably transduced cells shows near complete absence of VDR protein in JSC-1 cells. (**B** and **C**) Cell apoptosis was significantly decreased in JSC-1 cells with VDR knockdown as compared to control shRNA transduced cells. (**D**) Western blotting with anti caspase-3, anti ORF57 and β-actin antibodies of cell lysates of JSC-1 VDR KO cell line and JSC-1 Ctrl treated with 10 nM of 1, 25(OH)2 D3 and 20 ng/ml of TPA used as positive control.

To further confirm the role of VDR in KSHV reactivation, we also used lentiviral shVDR, a plasmid that express short hairpin RNA (shRNA) targeting VDR, to examine the effect of VDR depletion on KSHV reactivation. Table 1 shows the 2 shRNA constructs targeting 2 discrete regions of the human VDR transcript. We selected transfected cells with puromycin resistance gene to obtain stably transduced cells JSC-1-VDRKO. VDR knock-down JSC-1 cells were used in 1, 25(OH)2 D3 reactivation assays. Viral reactivation was measured by western analysis for the lytic protein, ORF-57. The control cell line, pTRCJSC-1 showed significant reactivation with 1, 25(OH)2 D3 (Fig. 6D) as compared VDR knockdown cells. Notably, all knock-down cell lines were responsive to lytic reactivation by TPA, and showed significant reactivation (Fig. 6D). In summary, VDR knock-down lowers 1, 25(OH)2 D3 induced viral reactivation.

## Discussion

In summary, we report here compelling evidence to KSHV reactivation through VDR signalling. VDR mediated reactivation from latency offers a paradigm for how KSHV may initiate lytic replication *in vivo*. The mechanisms controlling KSHV latncy and lytic replication are complex. Whether KSHV undergoes latent or lytic replication might depend on diverse factors including: the status of cellular signalling pathways, cell cycle, extracellular factors, cell types, stages of viral infection, and viral regulatory factors, and susceptibility to disease development^45–51^. Understanding the key cellular and molecular basis of KSHV latency and reactivation may provide newer control stategies. In this report, we demonstrate the involvement of VDR-dependent signal transduction in KSHV reactivation in latently infected cells.

The present work dissects the action of 1, 25(OH)2 D3 in PEL cells. Vitamin D receptor belongs to the superfamily of steroid receptors, which act as ligand dependent transcription factor. It is reported that VDR is constitutively expressed in primary effusion lymphoma B cells at high levels^11,52^. It has been previously demonstrated that active form of 1, 25(OH)2 D3 promotes growth inhibition in lymphocytes and in a variety of human cancer cell lines^53–57^. PEL cell line used in this study responds robustly to 1, 25(OH)2 D3 during a 48 h treatment period (Fig. 1). We observed strong growth inhibition at early time point from 24 h. However, this response was lost with incubation beyond 72 hours in JSC-1 cells (Fig. 1A). In contrast, a delayed response was noted in HBL-6 cells and growth inhibition starts 72 h post treatment (Fig. 1B). On the other hand, DG-75 cells showed no inhibition. Difference in sensitivity of these (PEL) cells to ligand may be due to the difference in receptor expression (Fig. 1C).

Reduction in growth, proliferation and induction of apoptosis are likely to cause Herpes virus reactivation^58–60^. Previous studies have shown that two seemingly conflicting phenotypes of KSHV reactivation and the death of PEL cells occur simultaneously^43^. Notably, 1, 25(OH)2 D3 mediated inhibition of proliferation was associated with apoptosis of the PEL cells (Fig. 2). On the other hand, the reactivation of PEL cells observed (expression of lytic transcripts ORF57 and K8.1) was not affected by pan caspase inhibitor (Fig. 3C and D) although it was able to suppress apoptosis (Fig. 3A), clearly indicating that these two actions are independent of each other. Even though the extent of reactivation by 1, 25(OH)2 D3 is comparatively lower than those caused by strong inducers, such as TPA. More importantly, unlike TPA and butyrate, 1, 25(OH)2 D3 is a natural product of cellular metabolism and plays a critical role in several physiological and pathological conditions. It is likely that 1, 25(OH)2 D3 may play a pivotal role in regulation and equilibrium between latent and lytic replication in PEL cells. Thus, our findings elucidate one of the possible mechanisms for the pathogenesis and reactivation associated with KSHV infection. To identify the mechanisms of reactivation of KSHV in PEL cells, several different signalling pathways have been investigated. Several authors have shown the involvement of MAPK pathways in 1, 25(OH)2 D3 treated cells. MAPK p38 has been shown to be involved skeletal and intestinal cells, thereby affecting cell cycle, growth and differentiation^61,62^. Previous reports have also shown the involvement of MAPK pathways in KSHV lytic replication during productive primary infection and reactivation from latency^63,64^. In this study, the p38 MAPK pathway however appears to be generally activated by 1, 25(OH)2 D3 in PEL cells as assessed by expression of p-p38 protein (Fig. 6A). Most importantly, this activation of p38 MAPK by 1, 25(OH)2 D3 led to reactivation of KSHV in PEL cells. Furthermore, the p38 kinase inhibitor SB203580 not only prevented p38 phosphorylation but also abrogated KSHV reactivation (Fig. 5B). On the other hand, ERK inhibitor, PD98059 only partially suppressed KSHV reactivation, while there was no change with JNK inhibitor (Fig. 5A and B) suggesting that MAPK pathways p38 and ERK may be involved in switching from latency to lytic phase in KSHV.

Treatment of 1, 25(OH)2 D3 in PEL cells caused p38, ERK expression and caspase activation indicating that signalling events bifurcate downstream of VDR in mediating these two processes, i.e., virus reactivation and cell death. KSHV reactivation from latency depends on the expression of RTA. Our finding mirrors this as RTA expression occurred following stimulation by 1, 25(OH)2 D3 (Fig. 4A and B). As RTA does not contain any VDRE, it is speculated that 1, 25(OH)2 D3 indirectly increases the RTA expression most likely via p38. Thus, increase in the RTA expression induced by 1, 25(OH)2 D3 may lead to a greater degree of KSHV reactivation, making RTA a sensitive regulator between latency and reactivation as also confirmed through infection of 293 cells (Fig. 4G).

Lastly, we determined the effects of VDR knockdown on PEL cell proliferation and KSHV reactivation. VDR Knockdown rendered JSC-1 cells significantly less susceptible to 1, 25(OH)2 D3 mediated KSHV reactivation, while virus reactivation by phorbol esters remained intact (Fig. 6D). The findings further suggest that 1, 25(OH)2 D3 may activate latent KSHV *in vivo*. Thus, our findings clearly establish a key role in which VDR signalling allows the virus to escape a cell that is destined to die and induces KSHV reactivation and lytic replication. These findings imply a cross talk between a host cell and a latent KSHV that determine the clinical consequences.
